# Comparison of sarcopenia screening indices using serum creatinine and cystatin C in metabolic dysfunction-associated steatotic liver disease

**DOI:** 10.3389/fmed.2025.1633837

**Published:** 2025-08-07

**Authors:** Inyoung Hwang, Shi-Ra Lee, Yun Kim, Sang Won Lee

**Affiliations:** ^1^Department of Clinical Pharmacology and Therapeutics, Hanyang University Seoul Hospital, Seoul, Republic of Korea; ^2^Department of Pharmacology, Hanyang University College of Medicine, Seoul, Republic of Korea; ^3^College of Pharmacy, Daegu Catholic University, Gyeongsan, Republic of Korea

**Keywords:** MASLD, sarcopenia, creatinine, cystatin C, muscle mass estimation

## Abstract

**Background:**

Metabolic dysfunction-associated steatotic liver disease (MASLD) and sarcopenia share underlying pathophysiological mechanisms and can bidirectionally influence the development and progression of each other. Diagnosing sarcopenia in individuals with MASLD is challenging due to overlapping symptoms and the frequent requirement for expensive, specialized equipment for muscle mass assessment. Therefore, accessible screening methods are crucial. Serum indices based on creatinine (Cr) and cystatin C (CysC), including Calculated Body Muscle Mass (CBMM), Sarcopenia Index (SI), and estimated glomerular filtration rate (eGFR) ratio, have emerged as potential biomarkers for sarcopenia. This study aimed to compare the performance of these serum indices in screening for low skeletal muscle index (SMI) among patients with MASLD.

**Methods:**

This prospective observational study enrolled 146 participants with MASLD. Anthropometric and laboratory data were collected. The CBMM, SI, and eGFR ratios were calculated using serum Cr and CysC levels. Low SMI was determined using Bioelectrical Impedance Analysis (BIA) according to the Asian Working Group for Sarcopenia (AWGS) 2019 criteria. Linear regression analysis was used to assess the correlations between serum indices and SMI. Receiver Operating Characteristic (ROC) curve analysis was used to evaluate the discriminative ability of these serum indices for detecting low SMI. Furthermore, machine learning models (Linear Regression, Random Forest, and XGBoost), coupled with SHapley Additive exPlanations (SHAP) analysis, were employed to evaluate the importance of these indices in predicting low SMI.

**Results:**

Patients with higher fibrosis-4 (FIB-4) scores (≥2.67) had a significantly higher prevalence of low SMI. CBMM demonstrated the strongest correlation with SMI (R^2^ = 0.4306, *p* < 0.0001) and the best discriminative ability for low SMI (Area under ROC: 0.9149 for males and 0.9444 for females) compared with SI and eGFR ratio. Machine learning models consistently identified CBMM as the most important feature for predicting SMI based on the SHAP analysis.

**Conclusion:**

These findings suggest that CBMM, derived from readily available serum markers, could serve as a valuable initial screening tool for identifying MASLD patients at risk of sarcopenia who may benefit from further assessment and early interventions.

## 1 Introduction

Metabolic dysfunction-associated steatotic liver disease (MASLD), previously known as non-alcoholic fatty liver disease (NAFLD), has emerged as a leading cause of liver disease worldwide, propelled by the global epidemic of metabolic syndromes including obesity and type 2 diabetes mellitus (1). The estimated global prevalence of MASLD in the adult population is 32%, which varies significantly by region and is higher in men compared to women (2). MASLD encompasses a spectrum of liver diseases, ranging from simple steatosis to metabolic dysfunction-associated steatohepatitis (MASH), formerly known as non-alcoholic steatohepatitis (NASH), and can lead to complications such as cirrhosis, end-stage liver disease, and hepatocellular carcinoma (3, 4). Projections indicate that the number of MASLD cases will continue to increase, imposing a considerable burden on the healthcare system (5). In the United States, hospitalization for MASLD-related decompensated cirrhosis and MASLD-related hepatocellular carcinoma showed 10.6 and 8% annual rate of increase, respectively, between 2005 and 2014 (6). Projections from 2016 estimated that over 64 million individuals would have MASLD, with associated annual medical costs totaling $103 billion (7). Risk factors for MASLD include genetic predisposition, demographic factors, health behaviors, and clinical factors (8). Among various clinical risk factors, sarcopenia is receiving increasing attention due to its shared underlying mechanisms and impact on the development and progression of MASLD (1).

Sarcopenia is defined as a progressive and generalized decline in skeletal muscle mass, strength, and physiological function (9). The prevalence of sarcopenia ranges from 8 to 36% in individuals aged < 60 years and from 10 to 27% in adults aged > 60 years (10). Although traditionally linked to the aging process, sarcopenia is now understood to be associated with other conditions such as metabolic syndrome, diabetes mellitus, cardiovascular diseases, inflammatory diseases, and chronic liver diseases (11, 12). Sarcopenia is prevalent in 40–60% of patients with end-stage liver disease (ESLD) and is associated with poor prognosis in this population (13). Therefore, both the relationship between chronic liver diseases and sarcopenia and strategies for the early diagnosis of sarcopenia in individuals with these conditions are receiving increased attention.

Bidirectional relationship between MASLD and sarcopenia has been reported by numerous observational studies (14). Sarcopenia has been identified as an independent risk factor for MASLD, increasing the risk by 2.3- to 3.3-fold (15). Conversely, in MASLD/MASH and cirrhosis patients, a higher prevalence of sarcopenia was observed compared to that in the general population (16). This interplay likely stems from shared pathophysiological mechanisms, including insulin resistance (IR), chronic inflammation, altered myokine secretion, imbalances of hormones, including growth hormone and IGF-1, vitamin D deficiency, and physical inactivity (15).

Despite its clinical significance, the identification of sarcopenia in patients with MASLD poses several challenges. First, in patients with MASLD, the representative symptoms of sarcopenia, such as muscle weakness and reduced physical activity, can overlap with the symptoms of MASLD itself, thereby complicating the diagnosis of sarcopenia based on clinical presentation (17). In addition, as various consortia and expert groups have proposed differing definitions, criteria, assessment tools, and threshold values, diagnosing sarcopenia can vary among clinicians and researchers without consensus (17). Standard diagnostic methods for quantifying muscle mass, including computed tomography (CT), magnetic resonance imaging (MRI), dual-energy X-ray absorptiometry (DXA), and bioelectrical impedance analysis (BIA), require specialized equipment and trained personnel and are associated with significant costs or exposure to radiation (18, 19). Therefore, there is a need for a simple, inexpensive, and readily accessible screening method that can be easily utilized in routine clinical settings for MASLD patient care to identify those at a high risk of sarcopenia.

Serum indices based on creatinine (Cr) and cystatin C (CysC) have been proposed as potential biomarkers for development and progression of sarcopenia (20). Cr is an endogenous substance derived from creatine and creatine phosphate produced in the skeletal muscle. As its production is relatively constant with stable muscle mass and excreted in the kidney primarily by filtration, creatinine has been routinely used to estimate glomerular filtration rate (eGFR), although its production, and hence eGFR_Cr_, may vary with body composition, such as elevated or reduced muscle mass (21). In contrast, CysC is constantly produced by all nucleated cells, and its production as well as eGFR_CysC_ are less affected by muscle mass (22). Therefore, serum indices combining Cr and CysC stem from leveraging their differential relationships with renal function and body composition. One of the indices is the sarcopenia index (SI), calculated as the ratio of serum Cr to serum CysC, which was able to estimate muscle mass among ICU patients (23). The eGFR ratio, defined as the ratio of eGFR_CysC_ to eGFR_Cr_, has been suggested as a screening marker for sarcopenia in community-dwelling older adults (24). Another index, calculated body muscle mass (CBMM), derived from serum Cr, CysC, body weight, and sex, has been introduced to estimate the total body muscle mass (25).

The existence of these different formulations underscores that no single Cr/CysC-based index has achieved universal acceptance or has demonstrated consistent superiority across all clinical contexts. We compared the predictive performance of the CBMM, SI, and eGFR ratio as screening indices of sarcopenia in patients with MASLD.

## 2 Methods

### 2.1 Study participants

This prospective, observational clinical study was conducted at Hanyang University Seoul Hospital between November 2021 and August 2022. Korean adults aged 19 years or older diagnosed with MASLD were eligible for inclusion in this study. The diagnosis of MASLD, termed NAFLD during the study period, was performed according to the 2021 guideline for the management of NAFLD from the Korean Association for the Study of the Liver (KASL) (26). A diagnosis of NAFLD was established based on evidence of hepatic steatosis (identified via abdominal ultrasonography, CT, MRI, or liver biopsy) and the exclusion of other causes including significant alcohol consumption, viral hepatitis, and the use of medications known to induce fatty liver. Participants diagnosed with chronic hepatitis other than MASLD, treated with high-dose corticosteroids (equivalent to prednisolone 20 mg/day) for over 14 days, or taking cyclosporin within 1 month prior to the screening visit were excluded from the study. Based on a previous study that compared serum indices for the prediction of sarcopenia in approximately 300 patients with liver disease, a target sample size of 150 participants with MASLD was established (27). All participants provided written informed consent before participating in the study. The study was approved by the Institutional Review Board of Hanyang University Seoul Hospital (IRB number: HYUH-2021-08-066) and was conducted in accordance with Korean Good Clinical Practice (KGCP) and the Declaration of Helsinki.

### 2.2 Measurements

Anthropometric and laboratory measurements were conducted for each participant during routine hospital visits. Laboratory examinations included aspartate transaminase (AST), alanine transaminase (ALT), platelet count, and serum Cr and CysC levels.

Three creatinine-and cystatin C-based indices (CBMM, SI, and eGFR ratio) were calculated as follows (23–25, 28, 29):

1.
$\text{CBMM}=\frac{\text{Body weight}\;[\mathrm{kg}]\times \text{Serum}\;\mathrm{Cr}\;[\mathrm{mg}/\mathrm{dL}]}{K\times \text{Body weight}\;[\mathrm{kg}]\times \text{Serum CysC}\;[\mathrm{mg}/L]+\text{Serum}\;\mathrm{Cr}\;[\mathrm{mg}/\mathrm{dL}]}\;$

where K = 0.00675 for males and K = 0.01006 for females.

1.
SI=SerumCr[mg/dL]/Serum CysC[mg/L]

2.
$\text{eGFR ratio}={\text{eGFR}}_{\text{CysC}}/{\text{eGFR}}_{\mathrm{Cr}}$

$\begin{array}{l} {\text{eGFR}}_{\mathrm{Cr}}=142\times \min{\left(\text{Serum}\;\mathrm{Cr}/\kappa ,1\right)}^{\alpha }\times \\ \max{\left(\text{Serum}\;\mathrm{Cr}/\kappa ,1\right)}^{-1.200}\times 0.9938^{\mathrm{Age}}\;[\times 1.012\;\text{if female}], \end{array}$ where κ is 0.7 for females and 0.9 for males, *α* is −0.241 for females and −0.302 for males.

$$\begin{array}{l} {\text{eGFR}}_{\text{CysC}}=133\times \min{\left(\text{Serum CysC}/0.8,1\right)}^{-0.499}\\ \times \max{\left(\text{Serum CysC}/0.8,1\right)}^{-1.328}\\ \times 0.9962^{\mathrm{Age}}\;[\times 0.932\;\text{if female}] \end{array}$$

The degree of liver fibrosis was estimated noninvasively using the FIB-4 score calculated as follows (30):

$$\begin{array}{l} \mathrm{FIB}-4=\mathrm{age}\;[\mathrm{yr}]\times \mathrm{AST}\;[U/L]\times \text{Platelet count}\\ {}[10^{9}/L]\times {\left(\mathrm{ALT}\;[U/L]\right)}^{1/2} \end{array}$$

Patients with FIB-4 scores ≥ 2.67 were classified into the high FIB-4 group, whereas those with scores < 2.67 constituted the low FIB-4 group (31).

### 2.3 Analysis of body composition

Bioelectric impedance analysis (BIA) was performed for each participant using an InBody 720 body composition analyzer (InBody Co., Ltd., Seoul, Republic of Korea) to assess body composition. The appendicular skeletal muscle mass (ASM), the sum of the lean muscle mass of the limbs, was adjusted for height to determine the skeletal muscle mass (SMI) as follows (32):

$$\mathrm{SMI}=\mathrm{ASM}\;[\mathrm{kg}]/\text{Height}\;{\left[m\right]}^{2}$$

Low SMI, indicative of low muscle mass for sarcopenia assessment, was defined using the criteria established by Asian Working Group for Sarcopenia (AWGS) 2019 criteria: <7.0 kg/m^2^ in men and <5.7 kg/m^2^ in women (19).

### 2.4 Statistical analysis

Continuous variables were expressed as mean (standard deviation), while categorical variables were expressed as number (%). The ratio of patients with low SMI was compared between the high FIB-4 group and low FIB-4 group using Fisher’s exact test. The correlation between serum indices (CBMM, SI, and eGFR ratio) and SMI was assessed using linear regression. Receiver operating characteristic (ROC) curve analysis was performed to evaluate the ability of each serum index to discriminate between patients with low and normal SMI. The area under the ROC curve (AUROC) with 95% confidence interval (CI) was calculated for each index. All statistical analyses were performed using SAS (version 9.4, SAS Institute Inc., Cary, NC, USA) and Prism (version 9.5.1, GraphPad Software, Boston, MA, USA). Statistical significance was defined as a two-sided *p*-value of < 0.05.

Machine learning (ML) models were developed to evaluate the predictive importance of serum indices for SMI. Three algorithms were selected: Linear Regression, Random Forest, and eXtreme Gradient Boosting (XGBoost). The input features for these models were CBMM, SI, and eGFR ratio, with SMI as the target variable. A 10-fold cross-validation procedure was implemented to train and validate the models, and the SHapley Additive exPlanations (SHAP) values were calculated to quantify the contribution of each input feature to the SMI prediction. For each model, the mean absolute SHAP value was determined for CBMM, SI, and eGFR ratio across all instances, thereby providing a global measure of feature importance.

Machine learning analyses were performed using Python (version 3.11.12, Python Software Foundation, Wilmington, DE, USA). The model implementation utilized the scikit-learn library (version 1.6.1) for Linear Regression, Random Forest Regressor, KFold cross-validation, and evaluation metrics. The XGBoost Regressor was implemented using the XGBoost library (version 2.1.4). SHAP values were computed using the shap library (version 0.47.2). Data manipulation and numerical operations were performed using Pandas (version 2.2.2) and NumPy (version 2.0.2), respectively. Figures were generated using Matplotlib (version 3.10.0).

## 3 Results

### 3.1 Participant characteristics

**Figure 1:**
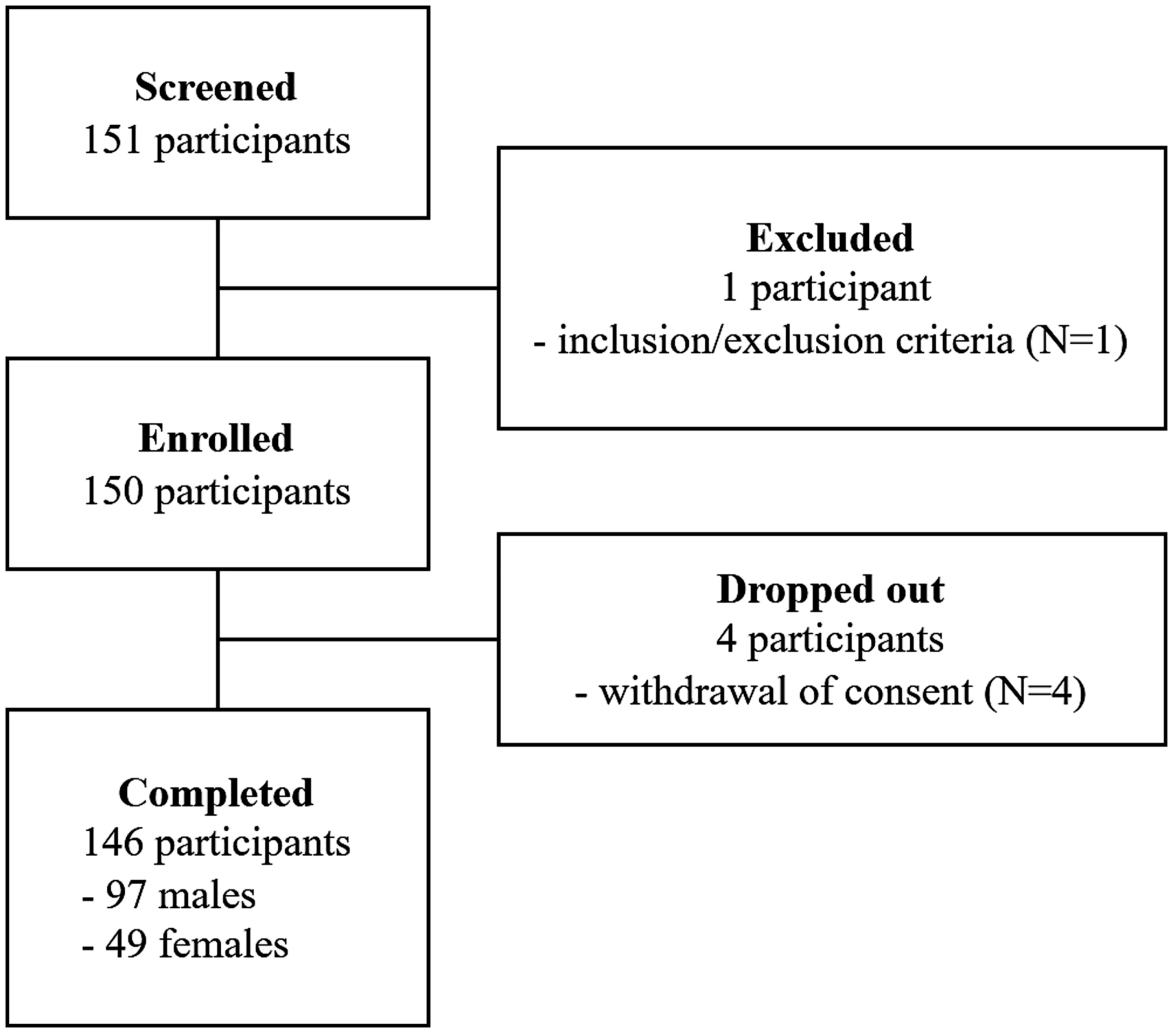
Disposition of study participants.

A total of 151 patients with MASLD were screened for eligibility, of whom 150 were enrolled in the study. Finally, 146 participants completed the study and were included in the final analysis (Figure 1). The baseline characteristics of the study participants are summarized in Table 1.

**Table 1:** Baseline characteristics of study participants.

Variable	All (*N* = 146)	Sex			SMI		
		Male (*N* = 97)	Female (*N* = 49)	*p*-value	Normal SMI (*N* = 139)	Low SMI (*N* = 7)	*p*-value
Age (years)	48.87 ± 15.58	45.94 ± 15.46	54.67 ± 14.25	0.0012	48.56 ± 15.41	55.00 ± 18.89	0.2875
Male [*n* (%)]	97 (66.44)	–	–	–	94 (67.63)	3 (42.86)	0.2244
Height (cm)	168.08 ± 9.31	172.82 ± 6.61	158.69 ± 6.22	<0.0001	168.54 ± 9.15	159.00 ± 8.04	0.0077
Weight (kg)	78.06 ± 16.24	83.28 ± 15.37	67.72 ± 12.65	<0.0001	79.32 ± 15.53	52.91 ± 7.16	<0.0001
BMI (kg/m^2^)	27.49 ± 4.39	27.82 ± 4.34	26.85 ± 4.46	0.2098	27.82 ± 4.21	20.93 ± 2.14	<0.0001
Creatinine (mg/dL)	0.83 ± 0.19	0.91 ± 0.16	0.66 ± 0.10	<0.0001	0.83 ± 0.19	0.77 ± 0.21	0.3765
Cystatin C (mg/L)	0.79 ± 0.18	0.80 ± 0.18	0.79 ± 0.18	0.7269	0.80 ± 0.18	0.77 ± 0.12	0.6937
AST (IU/L)	45.04 ± 33.45	46.22 ± 29.80	42.71 ± 39.96	0.5521	43.40 ± 26.52	77.57 ± 97.84	0.0079
ALT (IU/L)	48.37 ± 51.07	53.02 ± 48.24	39.16 ± 55.62	0.1220	47.19 ± 44.25	71.71 ± 131.63	0.2163
Platelet count (10^9^/L)	248.86 ± 74.81	253.41 ± 78.50	239.84 ± 66.74	0.3021	248.97 ± 74.99	246.57 ± 76.84	0.9343
FIB-4	1.70 ± 1.24	1.50 ± 1.15	2.11 ± 1.31	0.0045	1.66 ± 1.21	2.54 ± 1.53	0.0678
SMI (kg/m^2^)	7.99 ± 1.41	8.56 ± 1.18	6.85 ± 1.10	<0.0001	8.09 ± 1.35	5.89 ± 0.69	<0.0001
CBMM (kg)	49.24 ± 10.73	55.41 ± 6.99	37.05 ± 4.59	<0.0001	49.89 ± 10.44	36.43 ± 8.49	0.001
Sarcopenia index	107.04 ± 25.81	117.61 ± 22.90	86.11 ± 17.03	<0.0001	107.30 ± 25.53	101.91 ± 32.69	0.5922
eGFR ratio	1.02 ± 0.17	1.06 ± 0.17	0.95 ± 0.15	0.0002	1.02 ± 0.16	1.09 ± 0.26	0.3247

The mean age of the participants was 48.87 ± 15.58 years. Females exhibited a higher mean age (54.67 ± 14.25 years) compared to males (45.94 ± 15.46 years). Mean height and weight were greater in males (172.82 ± 6.61 cm and 83.28 ± 15.37 kg, respectively) than in females (158.69 ± 6.22 cm and 67.72 ± 12.65 kg, respectively). However, Body Mass Index (BMI) did not differ statistically between males (27.82 ± 4.34 kg/m^2^) and females (26.85 ± 4.46 kg/m^2^). Serum creatinine levels were higher in males (0.91 ± 0.16 mg/dL) compared to females (0.66 ± 0.10 mg/dL), while other clinical laboratory parameters showed no significant differences. The Fibrosis-4 (FIB-4) score was higher in females (2.11 ± 1.31) than in males (1.50 ± 1.15). SMI and serum indices (CBMM, SI, and eGFR ratio) were all higher in males (8.56 ± 1.18 kg/m^2^, 55.41 ± 6.99 kg, 117.61 ± 22.90, and 1.06 ± 0.17, respectively) compared to females (6.85 ± 1.10 kg/m^2^, 37.05 ± 4.59 kg, 86.11 ± 17.03, and 0.95 ± 0.15, respectively).

When participants were stratified by SMI, the low SMI group exhibited significantly lower mean values for height (159.00 ± 8.04 cm versus 168.54 ± 9.15 cm), weight (52.91 ± 7.16 kg versus 79.32 ± 15.53 kg), BMI (20.93 ± 2.14 kg/m^2^ versus 27.82 ± 4.21 kg/m^2^), SMI (5.89 ± 0.69 kg/m^2^ versus 8.09 ± 1.35 kg/m^2^), and CBMM (36.43 ± 8.49 kg versus 49.89 ± 10.44 kg) compared to the normal SMI group. However, SI and eGFR ratio were not statistically different between the low SMI group (101.91 ± 32.69 and 1.09 ± 0.26, respectively) and the normal SMI group (107.30 ± 25.53 and 1.02 ± 0.16, respectively). Aspartate Aminotransferase (AST) levels were significantly higher in the low SMI group (77.57 ± 97.84 IU/L) than in the normal SMI group (43.40 ± 26.52 IU/L).

### 3.2 Proportion of patients with low SMI in relation to FIB-4 score

**Figure 2:**
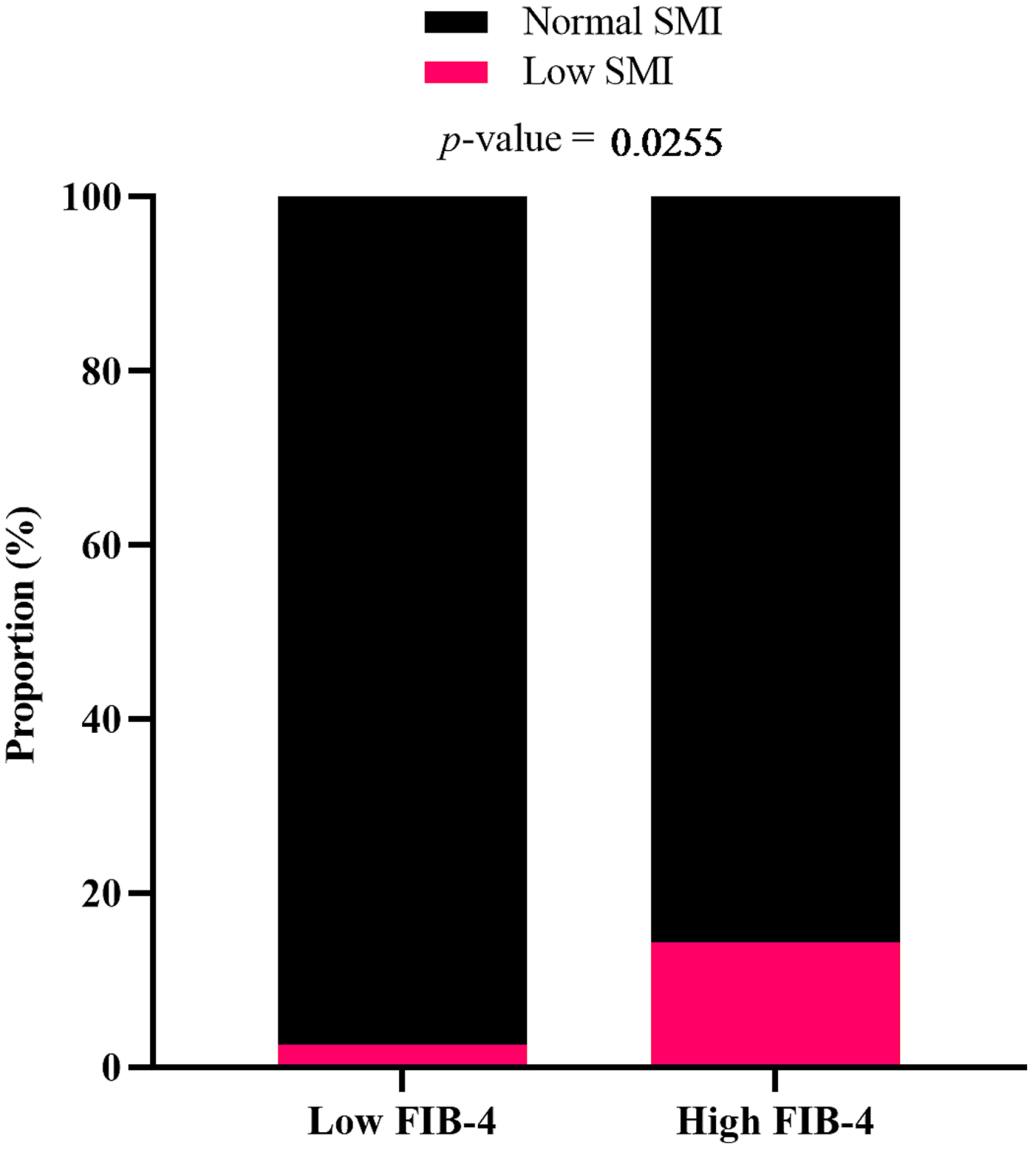
Proportion of MASLD patients with low SMI in relation to FIB-4 score. Participants were categorized into low FIB-4 score (<2.67) and high FIB-4 score (≥ 2.67) groups. *p*-value was determined by Fisher’s exact test.

The proportions of patients with low SMI were compared between the low FIB-4 score (<2.67) group and the high FIB-4 score (≥2.67) group, as shown in Figure 2. The proportion of participants with low SMI was significantly higher in the high FIB-4 score group. Similar trend was observed when participants were analyzed separately by sex, although the differences between groups were not statistically significant (Supplementary Figure 1).

### 3.3 Correlation between serum indices and SMI

**Figure 3:**
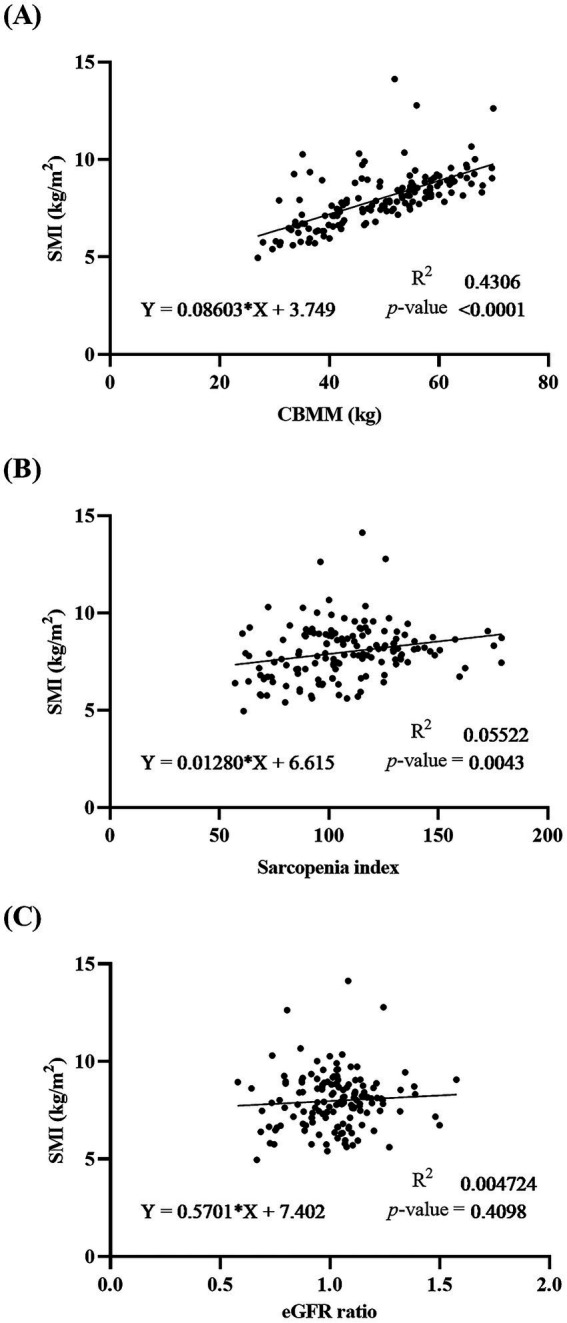
Linear regression analysis showing the correlation between **(A)** CBMM, **(B)** SI, and **(C)** eGFR ratio with SMI in patients with MASLD. Regression line equations, coefficients of determination (R^2^), and *p*-values are displayed for each panel.

The correlation between serum indices (CBMM, SI, and eGFR ratio) and SMI was analyzed using linear regression (Figure 3). Statistically significant correlation was observed in CBMM (R^2^ = 0.4306 and *p* < 0.0001), while no significant correlation was identified in SI and eGFR ratio (R^2^ = 0.0552 and *p* = 0.0043 for SI; R^2^ = 0.0047 and *p* = 0.4098 for eGFR ratio). Stratification by sex yielded similar results (Supplementary Figure 2).

### 3.4 Discriminative ability of serum indices in predicting SMI

The ability of the serum indices to discriminate low SMI from normal SMI was analyzed using the ROC curve (Table 2). CBMM demonstrated higher AUROC (0.9149 for male and 0.9444 for female) over SI (0.5532 for male and 0.5167 for female) and eGFR ratio (0.6489 for male and 0.6000 for female). Additionally, an alternative definition of low SMI (first quartile) was used to supplement this comparison. Similar to the low SMI definition of the AWGS 2019 criteria, the AUROC of CBMM was superior (0.8386 for male and 0.8243 for female) to SI (0.5924 for male and 0.5405 for female) and eGFR ratio (0.5228 for male and 0.5901 for female).

**Table 2:** Area under receiver operating characteristics curves (AUROC) and 95% confidence intervals (CI) for the prediction of SMI using CBMM, SI, and eGFR ratio.

Criteria for sarcopenia	Serum indices	Male (*N* = 97)		Female (*N* = 49)	
		AUROC	95% CI	AUROC	95% CI
AWGS 2019 criteria	CBMM	0.9149	0.8180–1.0000	0.9444	0.8704–1.0000
	SI	0.5532	0.0428–1.0000	0.5167	0.1551–0.8782
	eGFR Ratio	0.6489	0.3055–0.9924	0.6000	0.1781–1.0000
First quartile	CBMM	0.8396	0.7541–0.9252	0.8243	0.6897–0.9590
	SI	0.5294	0.3875–0.6713	0.5405	0.3343–0.7468
	eGFR ratio	0.5228	0.3824–0.6633	0.5901	0.3696–0.8106

### 3.5 Assessing the impact of serum indices on SMI using machine learning

**Figure 4:**
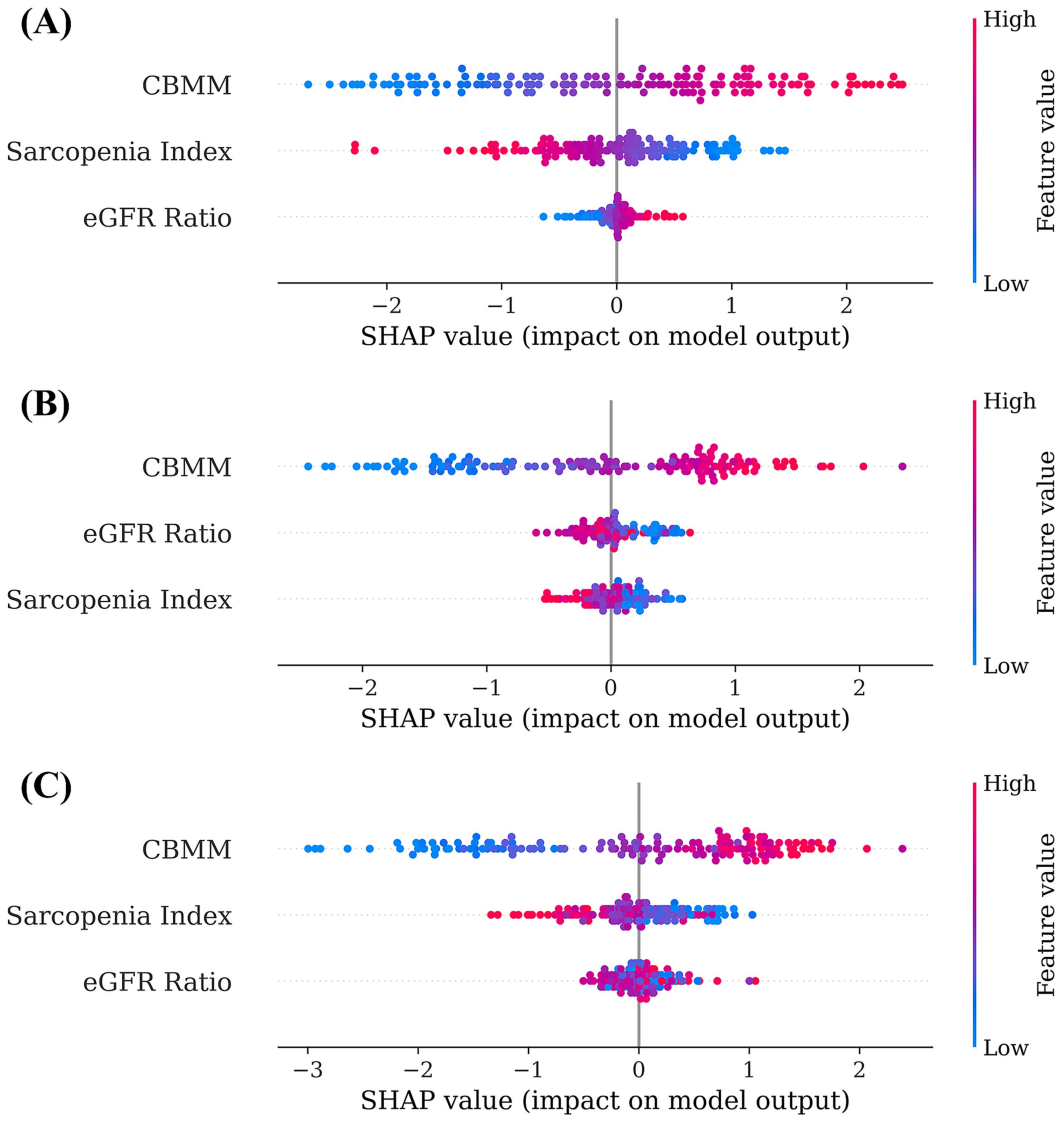
SHAP summary plots illustrating feature importance and impact for **(A)** linear regression, **(B)** random forest, and **(C)** XGBoost models predicting SMI.

**Figure 5:**
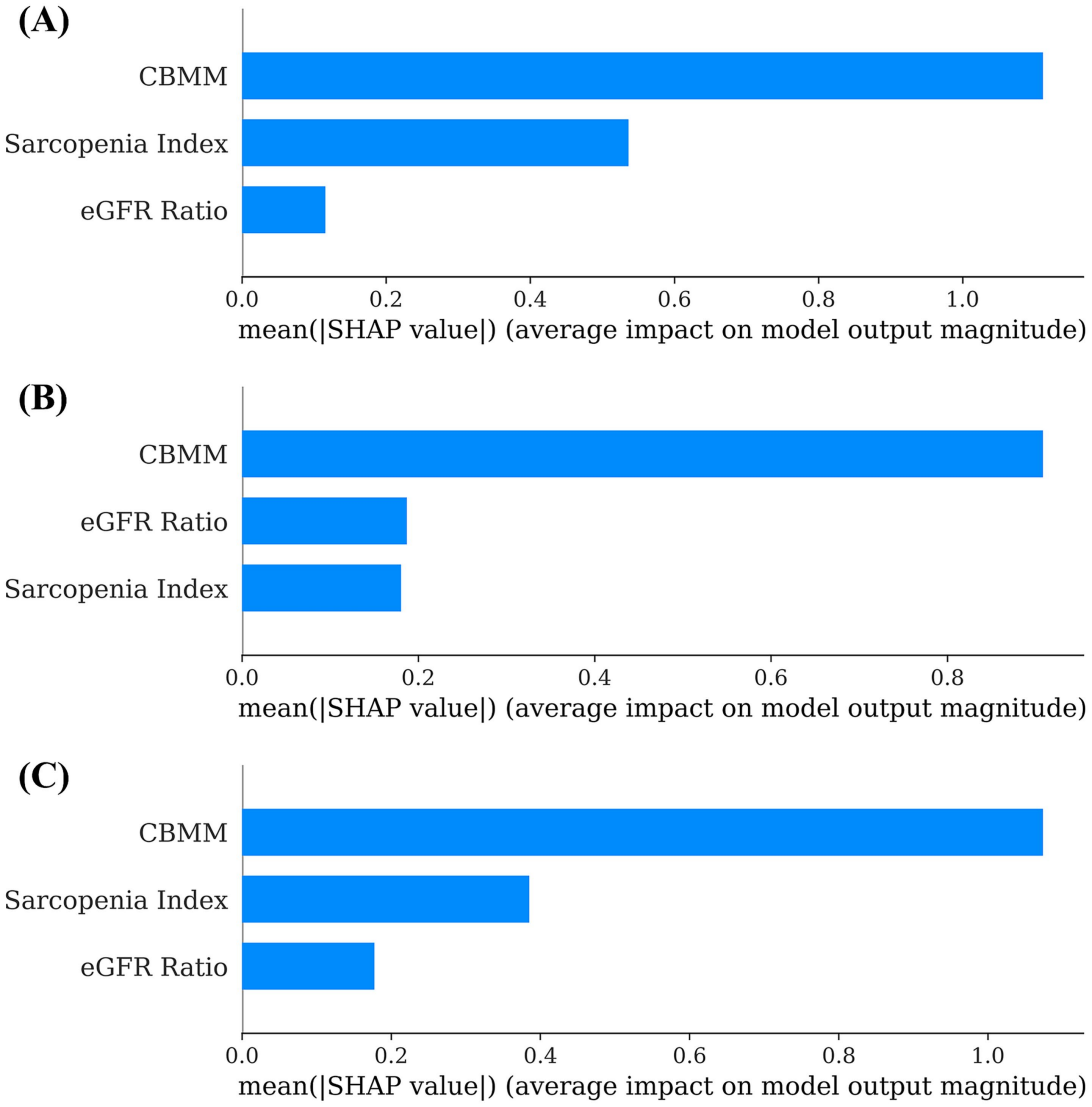
Global feature importance based on mean SHAP values for **(A)** linear regression, **(B)** random forest, and **(C)** XGBoost models predicting SMI.

Machine learning models (Linear Regression, Random Forest, and XGBoost) were developed to predict SMI using CBMM, SI, and eGFR ratio as input features. The predictive accuracy of these models was quantified using the following standard regression metrics: Mean Absolute Error (MAE), Mean Squared Error (MSE), Root Mean Squared Error (RMSE), and coefficient of determination (R^2^) (Table 3). The interpretability of these models was subsequently assessed using SHAP (SHapley Additive exPlanations) analysis to determine the relative importance of each input feature. Across all three models, CBMM consistently emerged as the most influential feature for predicting SMI, as visually illustrated in the SHAP summary plots (Figure 4). Specifically, the mean absolute SHAP values, which quantify the average impact of each feature on the model output, were highest for CBMM compared to SI and eGFR ratio (Figure 5). These findings from machine learning corroborate the results from traditional statistical analyses, providing robust evidence for the superior predictive value of CBMM for SMI in this MASLD cohort.

**Table 3:** Regression metrics of linear regression, random forest, and XGBoost models for predicting SMI.

Model metrics	Linear regression	Random forest	XGBoost
MAE	0.650 (0.169)	0.675 (0.191)	0.729 (0.221)
MSE	1.022 (0.855)	1.228 (0.945)	1.285 (0.992)
RMSE	0.936 (0.382)	1.032 (0.403)	1.053 (0.419)
R^2^	0.424 (0.346)	0.288 (0.456)	0.247 (0.505)

## 4 Discussion

This prospective observational study investigated the utility of serum indices based on creatinine and cystatin C to screen for low muscle mass, a key component of sarcopenia, in patients with MASLD. The principal findings were threefold. First, a higher risk of liver fibrosis, as indicated by an elevated FIB-4 score (≥2.67), was significantly associated with an increased prevalence of low SMI in this cohort. Second, when comparing the performance of the three Cr/CysC-based serum indices for prediction of SMI, CBMM demonstrated superior performance. CBMM exhibited the strongest correlation (highest R^2^) and the best discriminative ability (highest AUROC) compared to SI and eGFR ratio. Third, this superiority was further substantiated using machine learning models (Linear Regression, Random Forest, and XGBoost), where the SHAP analysis consistently identified CBMM as the most important feature among the three indices for predicting SMI.

The observed association between higher FIB-4 scores and increased prevalence of low SMI aligns with previous literature, indicating a link between the severity of MASLD and sarcopenia (33, 34). Histologic severity of MASLD has been identified to be associated with sarcopenia, and the complication rate of sarcopenia increased as the fibrosis progresses in MASLD/MASH patients (35, 36). Moreover, in a longitudinal study, MASLD patients with a high fibrosis risk, as indicated by NAFLD fibrosis scores (NFS) and FIB-4 scores, experienced a more rapid loss of skeletal muscle mass (36, 37). Several shared mechanisms may explain the observed association between MASLD and sarcopenia. Insulin resistance is a primary pathological mechanism causing both MASLD and sarcopenia, initiating from skeletal muscle loss and leading to increased lipolysis and dysregulation of the GH/IGF-1 axis (38). This process induces compensatory hyperinsulinemia, which promotes ectopic fat accumulation in the muscle and liver through altered glucose and lipid metabolism while also impairing protein synthesis and mitochondrial function, thereby exacerbating muscle degradation (39, 40). Sarcopenia and myosteatosis can further aggravate IR and glycemic dysregulation (41). Another pivotal factor in pathogenesis of MASLD is chronic inflammation and oxidative stress. Proinflammatory cytokines such as tumor necrosis factor-*α* (TNF-α) and transforming growth factor-*β* (TGF-β), coupled with reactive oxygen species (ROS) generated during fatty acid oxidation in the liver, induce chronic inflammation (15). Sustained inflammation contributes to liver injury and fibrosis progression (39). Furthermore, these cytokines promote protein catabolism, leading to a reduction in muscle mass and development of sarcopenia (42). Other mechanisms, such as vitamin D deficiency, dysregulation of myokines, and physical inactivity also contribute to the bidirectional relationship between MASLD and sarcopenia (14). However, a recent meta-analysis reported inconsistent findings regarding the association between sarcopenia and advanced fibrosis using the FIB-4 score (43). Therefore, while the current study supports an association between higher FIB-4 and low SMI in the MASLD group, caution is warranted before generalizing this finding.

The central finding of this study is the superior performance of the CBMM index compared to both the SI and eGFR ratio for identifying low SMI in patients with MASLD. This was evident in both stronger correlation to SMI and better discrimination of patients with low SMI. In a previous study on the utility of Cr/CysC-based indices in liver diseases, CBMM and SI were correlated with muscle mass, whereas difference in eGFR_Cr_ and eGFR_CysC_ (dGFR) was not. Moreover, the correlation coefficients of CBMM and SI with skeletal muscle mass were 0.804 and 0.293, respectively, indicating a strong correlation between CBMM and muscle mass (27). Couple of factors might explain the superior performance of CBMM in predicting muscle mass. First, the CBMM index was developed to estimate total body muscle mass (TBMM). Its derivation attempted to account for the relationship between Cr and muscle mass, and CysC and fat mass, after adjusting for eGFR (25). The final equation to estimate TBMM was developed after eliminating the eGFR and total body fat percentage. This modeling approach might have provided more accuracy to CBMM in the estimation of muscle mass compared to SI or eGFR ratio, which simply reflects the discrepancy between Cr/CysC or their derived eGFR as a surrogate for muscle mass. Second, as MASLD is often accompanied by metabolic diseases, such as obesity, and is characterized by altered body composition (visceral adiposity or sarcopenic obesity), an index designed to account for fat mass might have an advantage over other indices. The formulation of CBMM might more effectively isolate the muscle mass signal from this metabolic noise, especially in patients with MASLD.

The application of multiple machine learning algorithms (Linear Regression, Random Forest, and XGBoost) provided a robust framework for evaluating the relative importance of serum indices. The consistency of the findings across these models strengthens the conclusion that CBMM provides the most predictive information regarding SMI among the three indices studied. By quantifying the contribution of each index to the prediction of SMI for individual patients and averaging these contributions, SHAP provided evidence of CBMM’s superior feature importance reinforcing the results from traditional statistics.

The findings of current study suggest that CBMM could serve as a valuable initial screening tool for low muscle mass in routine MASLD clinical practice. Its primary advantage lies in its derivation from readily available and relatively inexpensive serum markers (Cr and CysC), making it far more accessible than imaging modalities, such as DXA or CT/MRI. While BIA is an existing accessible and relatively inexpensive method, it still requires a dedicated device, a scheduled patient visit, and trained personnel, which can present barriers to implementation at a population level. In contrast, since serum creatinine and cystatin C are frequently measured in routine metabolic panels for MASLD patients, CBMM can be calculated from existing electronic health record data, enabling large-scale surveillance without incremental costs or patient burden. Integrating CBMM calculation into clinical pathways could help clinicians identify MASLD patients who warrant further assessment for sarcopenia, including tests on muscle strength such as handgrip strength and physical performance, such as 5-time chair stand test. Additionally, confirmatory body composition analysis can be conducted if available. Early identification of sarcopenia could trigger timely interventions, such as tailored nutritional counseling and structured exercise programs, which are the cornerstones of sarcopenia management and may also benefit the MASLD itself.

The Cr/CysC-based indices are emerging as valuable tools for sarcopenia screening in various diseases beyond MASLD. For instance, one research group utilized the CBMM to assess the effect of direct-acting antivirals on muscle mass in patients with hepatitis C virus, a population often exhibiting low muscle mass and associated reduced quality of life (44). The Cr/CysC-based indices have also been employed to evaluate sarcopenia associated with chronic kidney disease (CKD) and non-small cell lung cancer (NSCLC) (45–47). However, the validity of the CBMM has not yet been established in patient populations other than those with chronic liver disease. Therefore, further studies are warranted to explore the application of the CBMM for assessing sarcopenia in a broader range of chronic diseases, as well as in primary muscle-wasting disorders such as Duchenne muscular dystrophy, to facilitate its versatile clinical utilization.

This study has strengths in the direct head-to-head comparison of three relevant Cr/CysC-based serum indices within an MASLD cohort and the application of interpretable machine learning methodology. However, there are limitations to this study that should be acknowledged. First, the sample size of 146 patients, while providing valuable initial data, was modest and may limit the generalizability of the findings. Therefore, additional studies with larger populations are necessary to validate the clinical utility of CBMM as a screening tool for sarcopenia in patients with MASLD. Second, although the data were collected prospectively, the analysis comparing serum indices in the prediction of SMI was cross-sectional. Further longitudinal studies are required to assess the prognostic value of these indices. Third, this study focused on low SMI and did not assess the functional aspects of sarcopenia, such as muscle strength or physical performance. Therefore, the indices were evaluated against a component of sarcopenia, not the clinical diagnosis of sarcopenia, according to the EWGSOP/AWGS criteria. Further studies should include these tests to assess the utility of these indices as screening tools for sarcopenia.

## 5 Conclusion

This prospective observational study compared the utility of Cr/CysC-based serum indices (CBMM, SI, and eGFR ratio) for screening low muscle mass in patients with MASLD, and found that CBMM demonstrated the strongest correlation with SMI and the best discriminative ability for low SMI among three indices. These findings suggest that CBMM could serve as a valuable initial screening tool for identifying MASLD patients at risk of sarcopenia who may benefit from further assessment and early interventions.

## Data Availability

The original contributions presented in the study are included in the article/Supplementary material, further inquiries can be directed to the corresponding authors.

## References

[1] Huh Y Cho YJ Nam GE. Recent epidemiology and risk factors of nonalcoholic fatty liver disease. J Obes Metab Syndr. (2022) 31:17–27. doi: 10.7570/jomes22021, PMID: 35332111 PMC8987457

[2] Riazi K Azhari H Charette JH Underwood FE King JA Afshar EE. The prevalence and incidence of Nafld worldwide: a systematic review and meta-analysis. Lancet Gastroenterol Hepatol. (2022) 7:851–61. doi: 10.1016/S2468-1253(22)00165-0, PMID: 35798021

[3] Vuppalanchi R Chalasani N. Nonalcoholic fatty liver disease and nonalcoholic Steatohepatitis: selected practical issues in their evaluation and management. Hepatology. (2009) 49:306–17. doi: 10.1002/hep.22603, PMID: 19065650 PMC2766096

[4] Muthiah MD Cheng Han N Sanyal AJ. A clinical overview of non-alcoholic fatty liver disease: a guide to diagnosis, the clinical features, and complications-what the non-specialist needs to know. Diabetes Obes Metab. (2022) 24:3–14. doi: 10.1111/dom.14521, PMID: 34387409

[5] Younossi ZM Golabi P Paik JM Henry A Van Dongen C Henry L. The global epidemiology of nonalcoholic fatty liver disease (Nafld) and nonalcoholic Steatohepatitis (Nash): a systematic review. Hepatology. (2023) 77:1335–47. doi: 10.1097/HEP.0000000000000004, PMID: 36626630 PMC10026948

[6] Kim D Cholankeril G Li AA Kim W Tighe SP Hameed B. Trends in hospitalizations for chronic liver disease-related liver failure in the United States, 2005-2014. Liver Int. (2019) 39:1661–71. doi: 10.1111/liv.14135, PMID: 31081997

[7] Younossi ZM Blissett D Blissett R Henry L Stepanova M Younossi Y. The economic and clinical burden of nonalcoholic fatty liver disease in the United States and Europe. Hepatology. (2016) 64:1577–86. doi: 10.1002/hep.28785, PMID: 27543837

[8] Iqbal U Perumpail BJ Akhtar D Kim D Ahmed A. The epidemiology, risk profiling and diagnostic challenges of nonalcoholic fatty liver disease. Medicine. (2019) 6:41. doi: 10.3390/medicines6010041, PMID: 30889791 PMC6473603

[9] Cruz-Jentoft AJ Bahat G Bauer J Boirie Y Bruyère O Cederholm T. Sarcopenia: revised European consensus on definition and diagnosis. Age Ageing. (2019) 48:16–31. doi: 10.1093/ageing/afy169, PMID: 30312372 PMC6322506

[10] Petermann-Rocha F Balntzi V Gray SR Lara J Ho FK Pell JP. Global prevalence of sarcopenia and severe sarcopenia: a systematic review and meta-analysis. J Cachexia Sarcopenia Muscle. (2022) 13:86–99. doi: 10.1002/jcsm.12783, PMID: 34816624 PMC8818604

[11] Li AA Kim D Ahmed A. Association of Sarcopenia and Nafld: An overview. Clin Liver Dis (Hoboken). (2020) 16:73–6. doi: 10.1002/cld.900, PMID: 32922754 PMC7474147

[12] Iwaki M Kobayashi T Nogami A Saito S Nakajima A Yoneda M. Impact of sarcopenia on non-alcoholic fatty liver disease. Nutrients. (2023) 15:891. doi: 10.3390/nu15040891, PMID: 36839249 PMC9965462

[13] Bhanji RA Carey EJ Yang L Watt KD. The long winding road to transplant: how sarcopenia and debility impact morbidity and mortality on the waitlist. Clin Gastroenterol Hepatol. (2017) 15:1492–7. doi: 10.1016/j.cgh.2017.04.004, PMID: 28400317

[14] Joo SK Kim W. Interaction between sarcopenia and nonalcoholic fatty liver disease. Clin Mol Hepatol. (2022) 29:S68. doi: 10.3350/cmh.2022.0358 36472051 PMC10029947

[15] Bhanji RA Narayanan P Allen AM Malhi H Watt KD. Sarcopenia in hiding: the risk and consequence of underestimating muscle dysfunction in nonalcoholic Steatohepatitis. Hepatology. (2017) 66:2055–65. doi: 10.1002/hep.29420, PMID: 28777879

[16] Zambon Azevedo V Silaghi CA Maurel T Silaghi H Ratziu V Pais R. Impact of sarcopenia on the severity of the liver damage in patients with non-alcoholic fatty liver disease. Front Nutr. (2021) 8:774030. doi: 10.3389/fnut.2021.774030, PMID: 35111794 PMC8802760

[17] Ye TD Mi K Zhu L Li J Pan CQ. Clinical characteristics of sarcopenia in nonalcoholic fatty liver disease: a systemic scoping review. Obes Facts. (2024) 18:1–14. doi: 10.1159/000541650, PMID: 39413746 PMC12017758

[18] Lee JH Kim HJ Han S Park SJ Sim M Lee KH. Reliability and agreement assessment of sarcopenia diagnosis through comparison of bioelectrical impedance analysis and dual-energy X-ray absorptiometry. Diagnostics. (2024) 14:899. doi: 10.3390/diagnostics14090899, PMID: 38732314 PMC11083379

[19] Chen LK Woo J Assantachai P Auyeung TW Chou MY Iijima K. Asian working Group for Sarcopenia: 2019 consensus update on sarcopenia diagnosis and treatment. J Am Med Dir Assoc. (2020) 21:300–7. doi: 10.1016/j.jamda.2019.12.012, PMID: 32033882

[20] Lian R Liu Q Jiang G Zhang X Tang H Lu J. Blood biomarkers for sarcopenia: a systematic review and meta-analysis of diagnostic test accuracy studies. Ageing Res Rev. (2024) 93:102148. doi: 10.1016/j.arr.2023.102148, PMID: 38036104

[21] Caregaro L Menon F Angeli P Amodio P Merkel C Bortoluzzi A. Limitations of serum creatinine level and creatinine clearance as filtration markers in cirrhosis. Arch Intern Med. (1994) 154:201–5. doi: 10.1001/archinte.1994.00420020117013, PMID: 8285815

[22] Levey AS Perrone RD Madias NE. Serum creatinine and renal function. Annu Rev Med. (1988) 39:465–90. doi: 10.1146/annurev.me.39.020188.002341, PMID: 3285786

[23] Kashani KB Frazee EN Kukralova L Sarvottam K Herasevich V Young PM. Evaluating muscle mass by using markers of kidney function: development of the sarcopenia index. Crit Care Med. (2017) 45:e23–9. doi: 10.1097/CCM.0000000000002013, PMID: 27611976

[24] Kusunoki H Tsuji S Kusukawa T Wada Y Tamaki K Nagai K. Relationships between cystatin C- and creatinine-based Egfr in Japanese rural community- dwelling older adults with sarcopenia. Clin Exp Nephrol. (2021) 25:231–9. doi: 10.1007/s10157-020-01981-x, PMID: 33090338 PMC7925493

[25] Kim S-w Jung H-W Kim C-H Kim K-i Chin HJ Lee H. A new equation to estimate muscle mass from creatinine and cystatin C. PLoS One. (2016) 11:e0148495. doi: 10.1371/journal.pone.0148495, PMID: 26849842 PMC4744004

[26] Kang SH Lee HW Yoo JJ Cho Y Kim SU Lee TH. Kasl clinical practice guidelines: management of nonalcoholic fatty liver disease. Clin Mol Hepatol. (2021) 27:363–401. doi: 10.3350/cmh.2021.0178, PMID: 34154309 PMC8273632

[27] Ichikawa T Miyaaki H Miuma S Motoyoshi Y Yamashima M Yamamichi S. Indices calculated by serum creatinine and cystatin C as predictors of liver damage, muscle strength and sarcopenia in liver disease. Biomed Rep. (2020) 12:89–98. doi: 10.3892/br.2020.1273, PMID: 32042417 PMC7006091

[28] Inker LA Schmid CH Tighiouart H Eckfeldt JH Feldman HI Greene T. Estimating glomerular filtration rate from serum creatinine and cystatin C. N Engl J Med. (2012) 367:20–9. doi: 10.1056/NEJMoa1114248, PMID: 22762315 PMC4398023

[29] Inker LA Eneanya ND Coresh J Tighiouart H Wang D Sang YY. New creatinine- and cystatin C-based equations to estimate Gfr without race. N Engl J Med. (2021) 385:1737–49. doi: 10.1056/NEJMoa2102953, PMID: 34554658 PMC8822996

[30] Sterling RK Lissen E Clumeck N Sola R Correa MC Montaner J. Development of a simple noninvasive index to predict significant fibrosis in patients with Hiv/Hcv coinfection. Hepatology. (2006) 43:1317–25. doi: 10.1002/hep.21178, PMID: 16729309

[31] Shah AG Lydecker A Murray K Tetri BN Contos MJ Sanyal AJ. Use of the Fib4 index for non-invasive evaluation of fibrosis in nonalcoholic fatty liver disease. Clin Gastroenterol Hepatol. (2009) 7:1104. doi: 10.1016/j.cgh.2009.05.033 19523535 PMC3079239

[32] Baumgartner RN Koehler KM Gallagher D Romero L Heymsfield SB Ross RR. Epidemiology of sarcopenia among the elderly in New Mexico. Am J Epidemiol. (1998) 147:755–63. doi: 10.1093/oxfordjournals.aje.a009520, PMID: 9554417

[33] Issa D Alkhouri N Tsien C Shah S Lopez R McCullough A. Presence of sarcopenia (muscle wasting) in patients with nonalcoholic Steatohepatitis. Hepatology. (2014) 60:428–9. doi: 10.1002/hep.26908, PMID: 24990106 PMC4539562

[34] Roh E Hwang SY Yoo HJ Baik SH Lee JH Son SJ. Impact of non-alcoholic fatty liver disease on the risk of sarcopenia: a Nationwide Multicenter prospective study. Hepatol Int. (2022) 16:545–54. doi: 10.1007/s12072-021-10258-8, PMID: 34780030

[35] Amer J Abdoh Q Salous Z Alsoud EA AbuBaker S Salhab A. A cross-sectional study of risk factors associated with sarcopenia in patients with metabolic dysfunction-associated Steatotic liver disease. Front Med. (2025) 12:1488068. doi: 10.3389/fmed.2025.1488068, PMID: 40041458 PMC11876153

[36] Demirci S Sezer S Erdoğan K Abdulsalam AJ Kara Ö Kara M. Strong association between Sarcopenic obesity and non-alcoholic fatty liver disease: An observational study with Isarcoprm algorithm. Clin Res Hepatol Gastroenterol. (2024) 48:102412. doi: 10.1016/j.clinre.2024.102412, PMID: 38964606

[37] Sinn DH Kang D Kang M Guallar E Hong YS Lee KH. Nonalcoholic fatty liver disease and accelerated loss of skeletal muscle mass: a longitudinal cohort study. Hepatology. (2022) 76:1746–54. doi: 10.1002/hep.32578, PMID: 35588190

[38] Kalyani RR Corriere M Ferrucci L. Age-related and disease-related muscle loss: the effect of diabetes, obesity, and other diseases. Lancet Diabetes Endocrinol. (2014) 2:819–29. doi: 10.1016/S2213-8587(14)70034-8, PMID: 24731660 PMC4156923

[39] Martin-Dominguez V Gonzalez-Casas R Mendoza-Jimenez-Ridruejo J Garcia-Buey L Moreno-Otero R. Pathogenesis, diagnosis and treatment of non-alcoholic fatty liver disease. Rev Esp Enferm Dig. (2013) 105:409–20. doi: 10.4321/s1130-01082013000700006, PMID: 24206551

[40] Turcotte LP Fisher JS. Skeletal muscle insulin resistance: roles of fatty acid metabolism and exercise. Phys Ther. (2008) 88:1279–96. doi: 10.2522/ptj.20080018, PMID: 18801860 PMC2579902

[41] Srikanthan P Hevener AL Karlamangla AS. Sarcopenia exacerbates obesity-associated insulin resistance and Dysglycemia: findings from the National Health and nutrition examination survey iii. PLoS One. (2010) 5:e10805. doi: 10.1371/journal.pone.0010805, PMID: 22421977 PMC3279294

[42] Phillips T Leeuwenburgh C. Muscle Fiber specific apoptosis and Tnf-alpha Signaling in sarcopenia are attenuated by life-long calorie restriction. FASEB J. (2005) 19:668–70. doi: 10.1096/fj.04-2870fje, PMID: 15665035

[43] Giri S Anirvan P Angadi S Singh A Lavekar A. Prevalence and outcome of sarcopenia in non-alcoholic fatty liver disease. World J Gastrointest Pathophysiol. (2024) 15:91100. doi: 10.4291/wjgp.v15.i1.91100, PMID: 38682026 PMC11045355

[44] Ichikawa T Miyaaki H Miuma S Motoyoshi Y Yamashima M Yamamichi S. Direct-acting antivirals improved the quality of life, ameliorated disease-related symptoms, and augmented muscle volume three years later in patients with hepatitis C virus. Intern Med. (2020) 59:2653–60. doi: 10.2169/internalmedicine.5102-20, PMID: 33132304 PMC7691030

[45] An JN Kim JK Lee HS Kim SG Kim HJ Song YR. Serum cystatin C to creatinine ratio is associated with sarcopenia in non-dialysis-dependent chronic kidney disease. Kidney Res Clin Pract. (2022) 41:580–90. doi: 10.23876/j.krcp.21.214, PMID: 35791742 PMC9576455

[46] Lin YL Wang CH Chang IC Hsu BG. A novel application of serum creatinine and cystatin C to predict sarcopenia in advanced Ckd. Front Nutr. (2022) 9:828880. doi: 10.3389/fnut.2022.828880, PMID: 35284461 PMC8914226

[47] Tang T Xie L Hu S Tan L Lei X Luo X. Serum creatinine and cystatin C-based diagnostic indices for sarcopenia in advanced non-small cell lung cancer. J Cachexia Sarcopenia Muscle. (2022) 13:1800–10. oi: 10.1002/jcsm.12977. doi: 10.1002/jcsm.12977, PMID: 35297568 PMC9178169

