# Supplementary material for: Comparison of sarcopenia screening indices using serum creatinine and cystatin C in metabolic dysfunction-associated steatotic liver disease
Source: Front Med (Lausanne). 2025 Aug 7;12:1633837. doi: 10.3389/fmed.2025.1633837 (PMC12367731; doi:10.3389/fmed.2025.1633837)
Supplement: Supplementary file 1 [file Data_Sheet_1.PDF]

Supplementary Material

1 Supplementary Figures and Tables

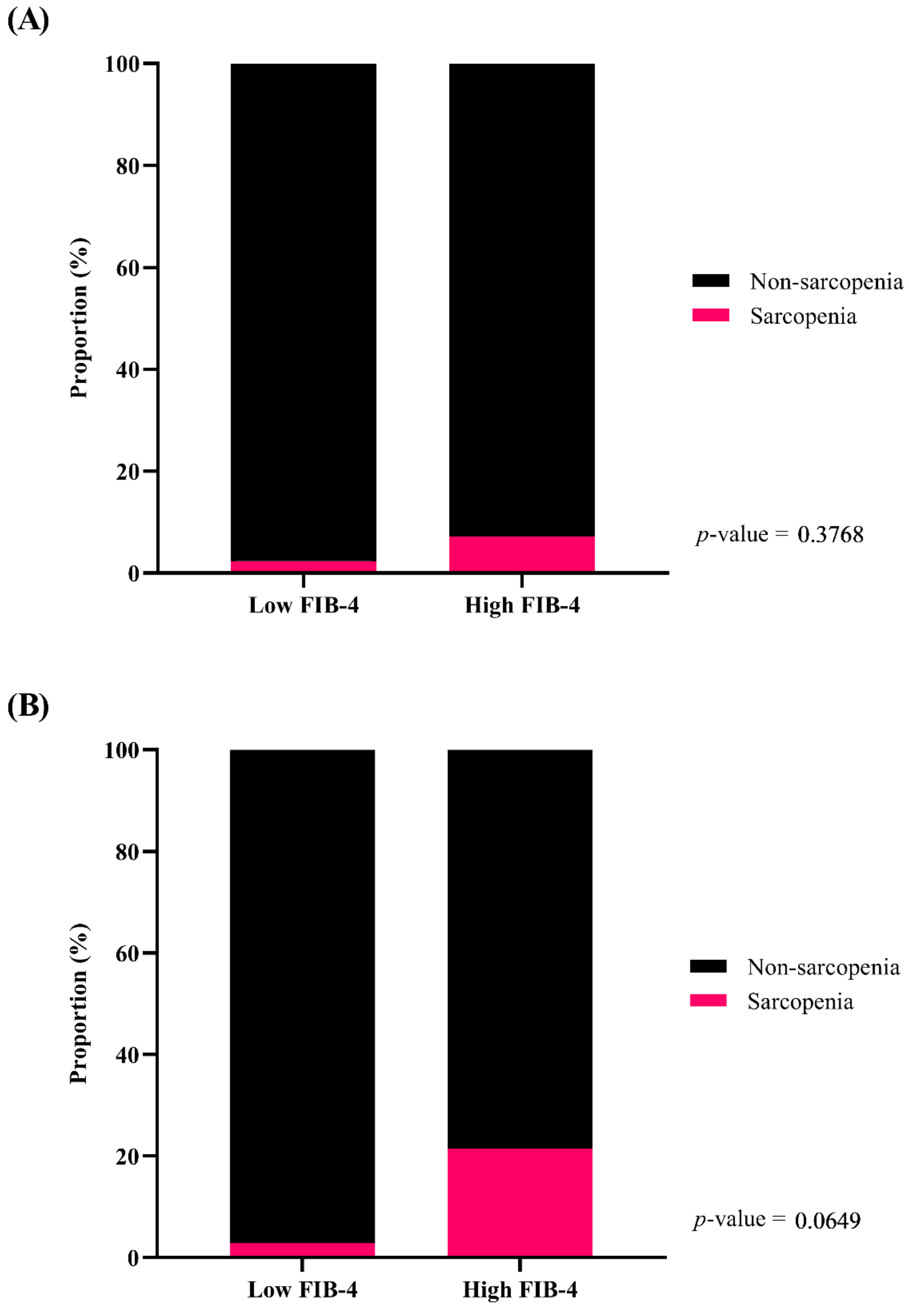

Supplementary figure 1. Proportion of (A) male and (B) female MASLD patients with low SMI in relation to FIB-4 score. Participants were categorized into low FIB-4 score (<2.67) and High FIB-4 score (≥2.67) groups. *p*-value was determined by Fisher's exact test.

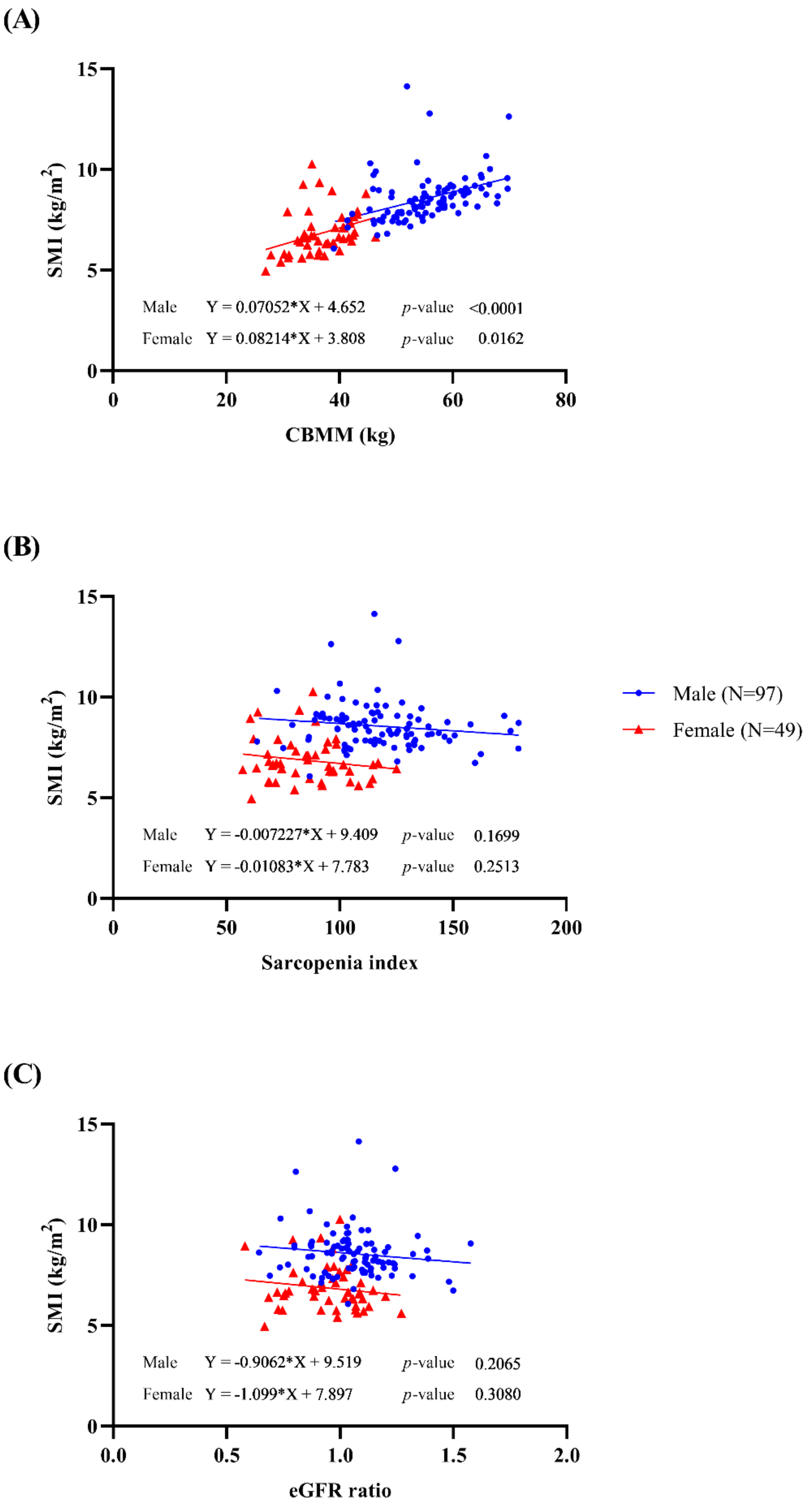

Supplementary figure 2. Linear regression analysis showing the correlation between (A) CBMM, (B) SI, and (C) eGFR ratio with SMI in male and female patients with MASLD. Regression line equations, coefficients of determination (R^2), and *p*-values are displayed for each panel.
